# New therapeutic targets for cancer: the interplay between immune and metabolic checkpoints and gut microbiota

**DOI:** 10.1186/s40169-019-0241-x

**Published:** 2019-08-27

**Authors:** Babita Agrawal

**Affiliations:** Department of Surgery, Faculty of Medicine and Dentistry, University of Alberta, Edmonton, AB Canada

**Keywords:** Tumor microenvironment (TME), Immune checkpoints, Metabolic checkpoints, Immune checkpoint inhibitors (ICIs), Monoclonal antibodies (mAbs), Gut microbiota

## Abstract

Transformation and growth of tumor cells are associated with profound alterations in neighbouring cells and their environment, together forming the tumor microenvironment (TME). The TME provides a conducive but complex milieu for the tumors to thrive while incapacitating the immune cells that home there as part of our natural immunosurveillance mechanism. The orchestration of this successful survival strategy by tumor cells is associated with exploitation of numerous metabolic and immune checkpoints, as well as metabolic reprogramming in the tumor cells. Together these form an intricate network of feedback mechanisms that favor the growing tumor. In addition, an ecosystem of microbiota, proximal or distal to tumors, influences the successful survival or elimination of tumor cells mediated by immune cells. Discovery and clinical application of immune checkpoint inhibitors (ICIs) i.e., monoclonal antibodies (mAbs) blocking specific immune checkpoints CTLA-4 and PD-1/PD-L1, have revolutionized therapy of various cancers. However, they are still associated with limited response rates, severe immune-related adverse events, development of resistance, and more serious exacerbation of cancer progression termed hyper-progressive disease. Checkpoint inhibitors only represent a milestone and not the finish-line in the quest for treating and curing cancer. Efforts are underway to investigate and develop inhibitors of other immune as well as metabolic checkpoint molecules. Future therapy for various cancers is projected to target immune and metabolic checkpoints and the microbiota together.

## Introduction

Cancer is among one of the leading causes of death worldwide with ~ 18 million new cases and ~ 9.5 million deaths in the year 2018 alone [1]. Furthermore, incidence of cancer is increasing worldwide and by 2030 it is expected to increase to ~ 24 million new cases each year [2]. Despite this, the overall cancer death rate in the United States fell by 26% from 1991 to 2015, due in part to screening programs for early detection and the advent of new therapeutic regimens [3]. Historically, chemotherapy, surgery and radiation therapy have been the mainstream cancer treatments. However, recent years have seen ground-breaking development of immunotherapies against various cancers, some have even emerged as first-line cancer therapies [4]. The idea of immunotherapy (therapeutic vaccines) for cancer has been intensely investigated in the last half century. However, most of the experimental therapeutic vaccine approaches for cancer tested clinically in the 1980s, 1990s and early 2000s did not prove to be efficacious, despite inducing sufficient and often robust immune responses [5]. Clearly, something was missing. The immune response generated systemically was either ineffective in reaching the tumor or was being modulated by the tumor environment resulting in the clinical inefficiency. As examples, tumors progressed even in the systemic presence of T cells and antibodies against tumor associated antigens (TAAs) such as carcinoembryonic antigen (CEA), MUC1 mucin (MUC1), human epidermal growth factor receptor-2 (Her-2Neu, also known as ErbB2), prostate specific antigen (PSA), melanoma antigens (MAGE-1, MAGE-2, MAGE-3) and others. This exposed the role of the tumor microenvironment (TME) in tumor progression and clinical failure of experimental vaccines [6]. Through intense research, it has now become apparent that the TME influences cancer progression and mortality, blunts immune responses and diminishes the efficacy of chemo- as well as immuno-therapeutics. Furthermore, TME has been shown to play a critical role in the development of advanced malignancies. The picture has become even more complex with the understanding of how the gut microbiota plays a role in tumorigenesis, malignancies and the response to various therapies. This review will provide a brief overview of the emerging concepts of immune and metabolic checkpoints, the tumor microenvironment and gut microbiota, and how they may be targeted clinically to achieve new milestones in cancer therapy.

### Cellular composition of the tumor microenvironment (TME)

**Fig. 1:**
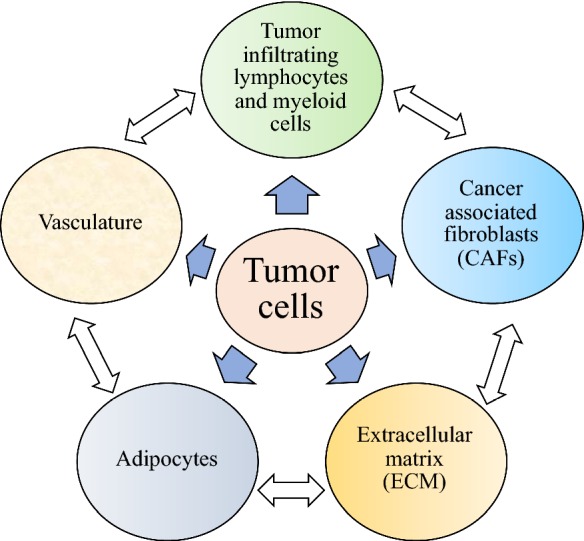
Composition of the tumor microenvironment (TME). The tumor microenvironment is composed of tumor cells, cancer-associated fibroblasts (CAFs), the extracellular matrix (ECM), adipocytes, tumor vasculature and tumor-infiltrating lymphocytes and myeloid cells, all of which cross-talk intensively through cell surface molecules and/or soluble mediators to promote tumor growth and protect from host immunity and/or therapeutics

Carcinogenesis or tumorigenesis, the formation of cancer, has three stages with dynamic and complex processes: initiation, progression and metastases. Although tumor formation is initiated by a transformed cell, there are multiple vital components which form an environment conducive for the tumor to grow and thrive. This is termed the tumor microenvironment (TME). The TME encompasses (i) tumor cells, (ii) cancer-associated fibroblasts (CAFs), (iii) the extracellular matrix (ECM), (iv) the tumor vasculature, (v) tumor infiltrating immune cells and (vi) adipose cells (Fig. 1), all of which have intensive cross-talk that promote tumor transformation, protect the tumor from host immunity, support tumor growth and invasion, and assist resistance of tumors to therapeutics [7].

The initial transformation of a normal somatic cell to a tumor cell is induced by genetic and epigenetic alterations, and/or cellular reprogramming, which lead to alteration in the control and activity of genes that regulate cell growth, proliferation and differentiation [8]. Tumor cells generally have an ability to evade growth suppressors, invade nearby tissues, produce energy in the absence of oxygen, resist apoptosis, hide from and modulate targets of drugs and the immune system, and have genomic instability [8].

Fibroblasts are mesenchymal in origin and found in every tissue of the body. Transiently activated fibroblasts, also termed as myofibroblasts, play an important role in tissue repair, extracellular matrix formation, wound healing and skin homeostasis [9]. During tumor formation, fibroblasts can also emerge by epithelial to mesenchymal transition (EMT) whereby epithelial cells that are adjacent to a transformed tumor cell lose cell–cell contact, develop mesenchymal properties and acquire invasive and migratory characteristics through genetic alterations [10]. A subpopulation of fibroblasts with a persistently activated phenotype are associated within the TME and are called cancer associated fibroblasts (CAFs) [11]. The signals mediating the transition of fibroblasts to CAFs are not yet clearly understood. It has been shown in cell culture that treatment of normal fibroblasts with TGF-β can induce a phenotype similar to CAFs [12]. The link between CAFs and tumor initiation and progression has been shown by comparing normal fibroblasts to CAF’s isolated from primary tumors. For example, Simian virus-40-induced normal prostate epithelial cells formed tumors in mice only when injected with CAFs [13]. Similarly, human breast cancer cell line MCF-7 formed bigger xenogeneic tumors in mice when injected with CAFs, compared to normal fibroblasts [10]. The mechanism of how CAFs promote tumor initiation and progression are not clear, however, they are capable of producing multiple soluble factors such as IL-1 and monocyte chemotactic protein-1 (MCP-1) (to induce inflammation), matrix metalloprotein (MMP) and vascular endothelial growth factor (VEGF) (to interact with microvasculature), transforming growth factor-β (TGF-β), hepatocyte growth factor (HGF) and stromal cell derived factor 1 (SDF1) (to induce tumor cell proliferation and invasion) [14].

The ECM components include collagen, laminins, fibronectins, proteoglycans and hyaluranans in a heterogeneous and complex organization [15]. The ECM influences cell invasiveness and cell metastasis. ECM provides a structural scaffold and contains soluble factors secreted by tumor cells, fibroblasts and immune cells in the vicinity. ECM also fosters the growth and survival of newly recruited endothelial cells and maintains the pH of the environment to become part of the TME [16].

The tumor vasculature is critical to the growth and sustenance of the tumor and its metastasis. Tumor angiogenesis allows the development of complex blood vessel and lymphatic networks within the TME so that there is supply of nutrients and a means to remove metabolic products. It also acts as a conduit for the transformed metastatic cells to leave and invade different tissues [17]. Some of the angiogenic factors include VEGF, basic fibroblast growth factor (bFGF) and interleukin-8 (IL-8), which together override the angiostatic signals such as angiostatin and endostatin [18]. However, the newly formed TME vasculature is leaky, immature, thin-walled and unorganized, contributing to maintain the hypoxic environment in the TME. The hypoxia induces tumor cells to increase anaerobic glycolysis, which in turn causes them to undergo genetic and epigenetic changes that make them more aggressive, and results in producing an acidic and immunosuppressive environment [19]. TME associated hypoxia has been suggested to induce a cancer stem cell (CSC)-like phenotype in tumor cells. Cells with this phenotype are undifferentiated with an extreme renewal potential that contributes to extreme tumor heterogeneity [20]. Most of the tumors are infiltrated with both helper and cytotoxic T cells, as well as innate immune cells, whose functions are blunted, allowing tumor cells to thrive even in their presence [21]. Oncogene-driven expression of IL-6, IL-10 and VEGF also promote a tolerogenic dendritic cell phenotype which negatively affects antitumor T cell responses [22].

In addition to immune suppression, recent studies demonstrate a significant role for inflammation in promoting tumorigenesis and tumor progression [23]. Persistent infection within the host results in low levels of chronic inflammation. Reactive oxygen and nitrogen species produced by phagocytes and leukocytes induce DNA damage in proliferating cells and form peroxynitrite, a mutagenic agent [24]. Some examples of inflammation-induced tumor progression include colon cancer arising in individuals with ulcerative colitis and Crohn’s disease, hepatocellular carcinoma associated with hepatitis C virus (HCV) and hepatitis B virus (HBV) infections, and stomach cancer associated with chronic *H. pylori* infection [25].

Adipocytes have been shown to play an active role in tumor growth in the TME [26]. Adipose tissues form 18–31% of the body mass in a normal healthy human [27]. White adipose tissue (WAT), besides serving as energy depot, are an active source of numerous soluble factors, termed adipokines, which include growth factors, hormones, cytokines, chemokines, leptin, and adiponectin. Many of the adipokines e.g., leptin, adiponectin, estrogen, insulin-like growth factor-1 (IGF-1) and hepatocyte growth factor (HGF), IL-6, and resistin, promote tumor growth. Excess adipocytes in the body lead to low-grade chronic inflammation, contributing to the development of cancer [28]. Cell culture studies have clearly demonstrated that co-culture of colon cancer, prostate cancer and melanoma cell lines with adipocytes promotes their proliferation [29]. Furthermore, co-culture with adipocytes led to enhanced migration and invasiveness of breast cancer cells [30]. However, contrasting studies demonstrating a negative role of adipocytes on tumor cells have also been reported [31].

In summary, multiple components of the TME contribute to tumor development and understanding their role, interaction and impact is essential to the development of novel therapeutic strategies.

### Immunometabolism in TME: metabolic checkpoints

**Fig. 2:**
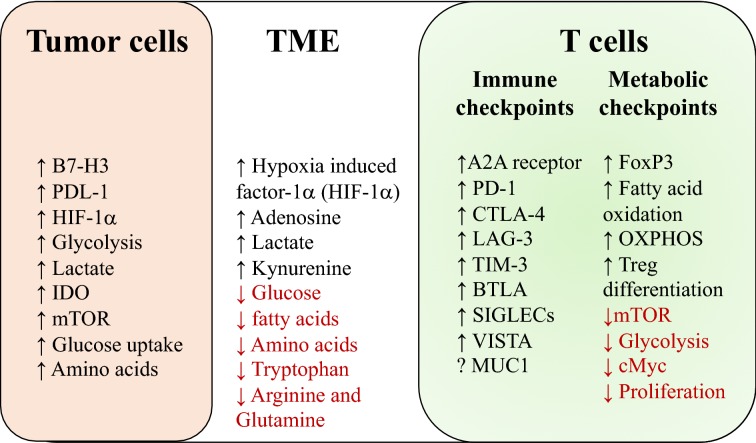
Immune-metabolism in the tumor microenvironment (TME). Fast growing tumor cells, under hypoxic conditions, lead to upregulation of HIF-1α, enhanced consumption of nutrients, increased glycolysis and increased production of adenosine and lactate, all of which in turn produce a microenvironment rich in adenosine, lactate and HIF-1α, but with a scarcity of nutrients for tumor-infiltrating immune cells. This metabolic microenvironment programs the immune cells such that (1) anergic T cells, with inefficient proliferation and effector function, as well as (2) regulatory T cells, are predominant in TME. Furthermore, T cells with increased expression of a number of immune checkpoints and loss of effector functions, a hallmark or exhausted T cells, are characteristics of TME. This figure depicts T cells as an example immune cell type for clarity, but as described in the manuscript text, other lymphoid and myeloid cells are also present in the TME and are intensively involved in tumor progression

Fast dividing tumor cells in hypoxic conditions, along with the TME, produce a metabolic environment which may significantly impact the functionality of immune cells in the TME (Fig. 2). This is mainly due to competition for nutrients and the production of numerous metabolites, some of them are described briefly as follows. Hypoxia results in upregulation of hypoxia-induced factor-1α (HIF-1α) and the expression of PD-L1 on tumor cells [32]. Hypoxia also leads to a high concentration of adenosine produced by tumor cells, which exerts an immunosuppressive function by directly binding to adenosine receptors (A2A) present on most immune cells [33]. Adenosine-mediated stimulation of A2A receptors leads to impaired T cell activation (reduced proliferation, cytokine production and cytotoxicity), compromised antigen presenting cell function (inhibited antigen uptake and reduced expression of MHC and co-stimulatory molecules), and reduced NK cell activation (cytokine production and cytotoxicity). It also induces the differentiation of myeloid-derived suppressor cells (MDSCs) and production of FoxP3 (associated with regulatory T cells (T_reg_ cells)), crippling almost all of the immune cells within the TME [34]. Hypoxia also inhibits T cells directly in an adenosine-independent manner [33]. Also within the TME, the scarcity of available glucose, fatty acids and amino acids results in impaired activation, differentiation and proliferation of T cells, which require high concentrations of these nutrients to sustain increased activity [35]. Tumor cell reprogramming towards glycolysis produces high amounts of lactate within the TME, which has a multifactorial impact on both tumor and immune cells within the TME. In an HIF-1α dependent pathway, lactate promotes vascular endothelial growth factor secretion and polarization towards M2 macrophages, which produce Arginase 1, promoting tumor proliferation and growth [36]. Further, lactate exerts a direct immunosuppressive effect on T and NK cells by directly impairing NFAT-1 (nuclear factor for activated T cells-1) resulting in reduced IFN-γ production [37]. In addition, low levels of arginine and glutamine in the TME prevent memory T cell formation and epigenetic modification in tumor cells, respectively, allowing optimum conditions for tumor immune evasion [38]. Tumor-generated indoleamine 2, 3-dioxygenase (IDO), metabolizes tryptophan, depleting the essential amino acid, tryptophan and producing kynurenines, which together create a profound immunosuppressive milieu within the TME that induces T cell anergy, and enhances proliferation and differentiation of T_reg_ [39].

T cell activation leads to a metabolic switch, with activation of a number of transcription factors and signaling pathways. T cell receptor together with CD28 co-stimulation activates 3-phosphoinositide-dependent protein kinase 1 (PI3K-1) and Akt, which in turn activate the central metabolic regulator mammalian target of rapamycin (mTOR). mTOR, comprised of mTORC1 and mTORC2, co-ordinates the increased requirement of nutrient levels, increased glycolysis and energy status of activated T cells to turn into effector cells. mTOR complex controls the transcription factors c-Myc and HIF-1α, which together activate the genes required for effector T cell proliferation. Interestingly, mTOR leads to downregulation of T_reg_ development, providing an important metabolic checkpoint of T cells [40]. In contrast, mTORC1 signaling is induced by innate toll like receptor (TLR) activation on T_reg_, which enhances the proliferation of T_reg_ and reduces their suppressive activity [41]. Foxp3, the transcription factor associated with T_reg_, reduces the effects of mTOR signaling to reduce glucose metabolism by glycolysis and, on the other hand, enhances oxidative phosphorylation (OXPHOS) to generate ATP from glucose and fatty acid oxidation (FAO) pathways [42]. The two distinct pathways of glycolysis versus OXPHOS and FAO appear to be central metabolic checkpoints to the control of T cell activation, effector function, and the fate of differentiating T cells. Hypoxia, and the nutrient deficit produced in the TME due to rapidly growing tumor cells, leads to inhibition of the mTOR pathway in T cells, resulting in inefficient proliferation, and reduced effector function and differentiation into T_reg_ [43].

There is also a reciprocal relationship between tumor metabolism and immune checkpoints. For example, it has been shown that B7-H3 (a putative immune checkpoint expressed on tumor cells and antigen presenting cells (APCs) in TME) plays an important role in regulating cancer cell metabolism, increases HIF-1α activity, glucose uptake, glycolysis and lactate production in breast cancer cells [44]. Furthermore, PD-L1 expressed in tumor cells promotes glycolysis via activation of the AKT/mTOR pathway [45]. Therefore, high expression of immune check point proteins in TME facilitates metabolic reprogramming in the tumor microenvironment, further providing a positive feedback loop to maintain and exacerbate the TME for the benefit of evolving tumor cells within. Tumor cells also have an exceptional ability to metabolically adapt to the changing environment in the TME due to scarcity of oxygen and reduced availability of nutrients. This gives rise to metabolic heterogeneity of tumor cells within the TME. Tumor metabolic heterogeneity could play a significant role in variable recruitment, activation, modulation and regulation of immune cells within the TME.

### Immunosuppression in TME: immune checkpoints

A startling revelation in tumor immunobiology was the realization that TME is not an immune-privileged site as once thought, but is rather infiltrated with a variety of innate and adaptive immune cells [46]. Further, the characteristics of immune cells within the TME often determine the fate with respect to tumor progression. Immune dysfunction within the TME has been characterized by accumulation of T cells with co-inhibitory receptors, antigen presenting cells with ligands for co-inhibitory receptors and T_regs_, which together function to allow tumor cells to evade immunity in a very prominent and successful manner.

The immune system in our body has evolved natural mechanisms, soluble mediators and receptor-ligand pairs, which can maintain immune homeostasis by keeping a check on aberrant immunity and allowing T cells to return to their normal resting stage after an antigenic stimulation. T cell responses are initiated through cellular interactions between APCs and T cells [47]. The type and strength of signals delivered through the T cell receptor (TCR) may modulate how the cells respond. However, the optimal T cell responses are dependent upon costimulatory and coinhibitory signals induced by interaction of various accessory cell surface molecules [48–50]. TCR engagement not only results in stimulation of T cells, its ligation also activates proteins (e.g., phosphatases), which serve as negative regulators of T cells and terminate T cell responses [51]. Direct inhibition of T cell proliferation or response is induced via signaling through two well-known negative regulators, also termed as co-inhibitory molecules or checkpoints, of T cells: cytotoxic T lymphocyte associated antigen 4 (CTLA-4) and programmed cell death 1 (PD-1), which play a crucial role in the maintenance of T cell homeostasis and peripheral tolerance [52]. Although both CTLA-4 and PD-1 are co-inhibitory molecules of T cells, they don’t have the same impact on immune homeostasis. Mice deficient in CTLA-4 have a lethal phenotype along with early onset of aggressive lymphoproliferative disorder. In contrast, PD-1 deficient mice survive and have organ-specific autoimmune diseases [53, 54]. Other T cell inhibitory molecules such as LAG-3 (lymphocyte activation gene 3), TIM-3 (T cell immunoglobulin and mucin domain 3), BTLA (B and T lymphocyte attenuator, CD272), SIGLECs 7 and 9 (Sialic acid binding Immunoglobulin-type lectin 7 and 9), VISTA (V-domain Ig suppressor of T cell activation), and A2A receptor (adenosine A2A receptor) are also checkpoint molecules, and function to limit ongoing immune responses [55]. Interestingly, many parallel inhibitory pathways have emerged through evolution that are expressed differentially on various subsets of T cells at different stages of activation. These have different functional impacts but work in concert to maintain self-tolerance and limit the scope of collateral damage upon induction of immunological responses in the peripheral system. The ultimate quality and quantity of T cell responses are regulated by a balance between costimulatory and coinhibitory (checkpoint) signals [51, 56]. CD4^+^ and CD8^+^ T cells expressing multiple immune checkpoint molecules during a normal response represent a phase of T cell activation [57].

It has been shown that continuous antigen exposure, along with other immunomodulating mechanisms in the TME, lead to exhausted T cells expressing high levels of co-inhibitory receptors (CTLA-4, PD-1, TIM-3, LAG-3, BTLA etc.) (Fig. 2) with loss of proliferation and effector function such as cytotoxicity, and production of effector cytokines (IFN-γ, IL-2, TNF-α) [58, 59]. However, T cell exhaustion is not a terminal differentiation state and is not irreversible, and by modulating the co-inhibitory pathways expressed by exhausted T cells, dysfunctional state of T cells can be reversed, forming the basis of current immune checkpoint inhibitor therapies [60].

Down-regulation of effector T cell function in TME is instigated and supported by APCs, which express increased levels of ligands for these co-inhibitory receptors like PD-L1, CD80/CD86, and B7H family molecules. Furthermore, the declining concentration of nutrients in the TME is directly associated with immune checkpoint expression. For example, lower glucose concentration directly upregulates PD-1 expression on T cells [61]. Interestingly, T_regs_, endowed with the ability to suppress effector T cell functions also express high levels of a number of co-inhibitory receptors (or checkpoints), and are prevalent in TME [62]. Therefore, successful survival and growth of tumor cells within our body is orchestrated by the TME as it exploits normal immune homeostatic mechanisms and steers them towards immune cells that express multiple immune checkpoints.

### An example of the complexity within the TME: MUC1 mucin as a tumor antigen, a metabolic checkpoint and an immune checkpoint

**Fig. 3:**
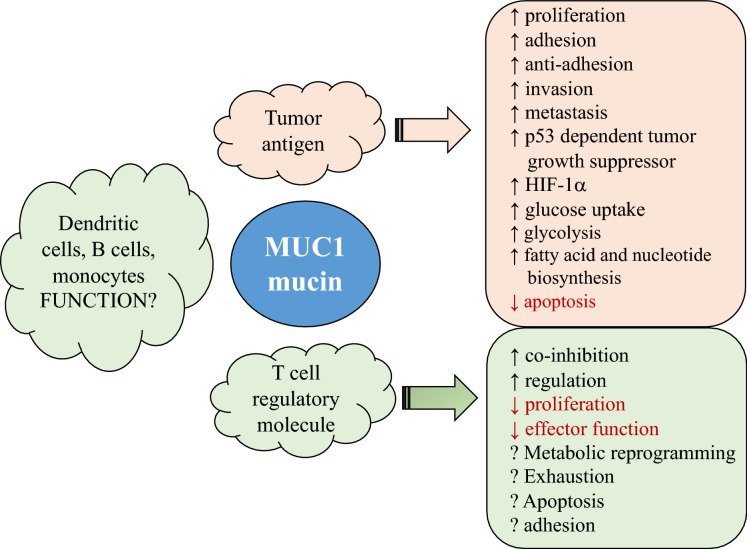
MUC1 mucin: a molecule with complex functions. MUC1 mucin can be characterized as a pan-tumor antigen, associated with adenocarcinomas, lymphomas and leukemias, where it plays a significant role in proliferation, metabolism and metastasis of tumor cells. In addition, MUC1 is also expressed on immune cells such as T cells, B cells, dendritic cells and monocytes. On T cells, MUC1 is putatively a checkpoint molecule and serves as a co-inhibitory molecule. The function of MUC1 on other immune cells is not clear yet. MUC1 mucin exemplifies the level of complexity present within the TME and consequently the development of cancer therapeutics

The level of complexity within the TME is impressive and at least partially contributes to the disappointing clinical responses associated with many of the current and experimental therapeutics. It is a formidable clinical challenge to determine how components of TME, tumor metabolism and immune checkpoints all come to play a role in successful survival and spread of tumors, despite the presence of all components of immunity in a host. A glimpse of this challenge is illustrated using the example of a ubiquitous adenocarcinoma tumor antigen MUC1 mucin as follows (Fig. 3).

MUC1 mucin, also known as polymorphic epithelial mucin (PEM) and CD227, is a large type 1 transmembrane glycoprotein, comprised of a variable number of extracellular tandem repeats (VNTR) of 20 amino acids each, a transmembrane region and a non-covalently attached cytoplasmic tail. The VNTR region and the whole protein is rich in serine and threonine, providing numerous sites for O-glycosylation. It also has some *N*-glycosylation sites in asparagine residues [63]. Hyperglycosylated MUC1 mucin is abundantly expressed on apical sites of normal secretory epithelial cells in respiratory, gastrointestinal, urogenital, and hepatobiliary tracts. MUC1 plays an essential role in forming mucus barriers that protect against infections and have both adhesive and anti-adhesive properties. However, aberrantly glycosylated, overexpressed MUC1 is a distinguishing feature of a large number of adenocarcinomas associated with lung, breast, pancreatic, ovarian, prostate, and colon cancers [64, 65]. MUC1 is also overexpressed in a number of hematological cancers, including B and T cell lymphomas and leukemias [66]. Therefore, MUC1 can be considered as a pan-cancer antigen. High levels of MUC1 expression and the presence of high concentrations of soluble MUC1 are associated with tumor progression and poor prognosis. The cytoplasmic tail of MUC1 is highly conserved and has been shown to bind to estrogen receptor alpha, p53, ErbB proteins, heat shock protein (Hsp)70, and Hsp90, making it a highly active cell signal transmitter [67]. MUC1 can interact with epithelial growth factor receptor (EGFR) and regulate Grb2/Sos/Ras-MEK-ERK2, and beta- and gamma-catenin signal pathways that are associated with proliferation, transformation, resistance to apoptosis, anti-adhesion to allow invasion, and metastasis [68]. MUC1 is an attractive target for a therapeutic cancer vaccine that could induce cellular and humoral immune responses.

Recent studies have demonstrated a new role of MUC1 as a metabolic modulator to reprogram tumor cells’ metabolism, mostly due to the exceptional signaling and transcriptional activity of its cytoplasmic tail (CT) [69]. The CT of MUC1 recruits transcription factor and co-factors to co-ordinate enhanced transcriptional activity of multiple genes by facilitating and co-occupying promoter/enhancer regions of the DNA [69]. MUC1-CT interacts with p53 to attenuate the p53-dependent tumor growth suppression and stabilizes HIF-1α to increase a number of effects: glucose uptake and glycolysis, nucleotide and fatty acid biosynthesis, direct regulation of tumor cell metabolic programming, and also allows the increased demand of nutrients and biosynthetic intermediates for fast dividing and metabolizing tumor cells [69].

Expression of MUC1 mucin was considered to occur strictly within epithelial cells until our original studies demonstrated that soluble cancer-associated MUC1 mucin can inhibit T cell responses and that activated human T cells also synthesize and express MUC1 mucin on their surfaces [70, 71]. We hypothesized that MUC1 mucin expressed by T cells plays an important role in regulating T cell responses, and in subsequent studies we confirmed that MUC1 can serve as a putative regulatory molecule of T cells, and is also expressed on majority of T_reg_ populations [72]. It remains to be examined whether MUC1 expressed by activated T cells leads to metabolic reprogramming of T cells as it does on tumor cells. The MUC1 cytoplasmic tail contains immunotyrosine-based inhibitory/activation motifs (ITIM/ITAM) sequences, supporting its function as a bona-fide co-inhibitory molecule of T cells like PD-1 [73]. Future investigation will determine how MUC1 expression may be modulated on T cells residing in TME. Besides activated T cells, MUC1 has been also shown to be expressed on other immune and hematopoietic cells (such as DCs, B cells, and monocytes), however, its function on these cells is still unclear [74].

### Microbiota and TME

**Fig. 4:**
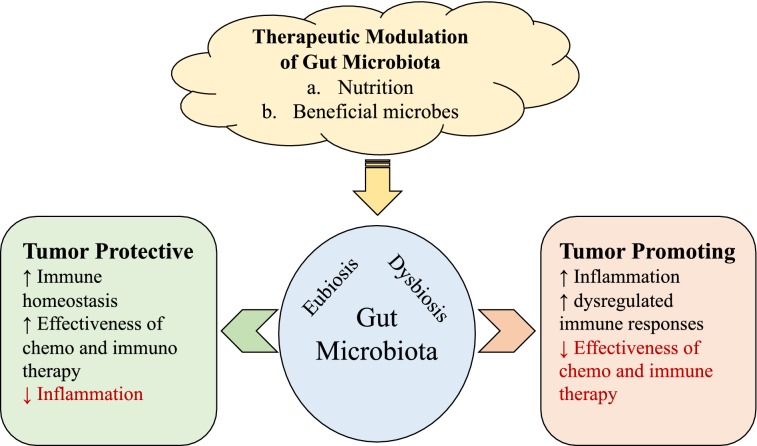
Role of gut microbiota in tumor progression/protection. Gut microbiota affects systemic inflammation, immune homeostasis and immune regulation. Therefore, eubiosis or dysbiosis of gut microbiota plays a significant role in protection from tumor growth or tumor progression, respectively. Furthermore, effectiveness of chemo- and immuno-therapy approaches is also at least partly governed by the state of gut microbiota. Modulation of gut microbiota through nutrition or ingestions of beneficial microbes may serve as important therapeutic modalities for cancer treatment alone or as an adjunct to chemo- and/or immuno-therapy

Microbiota has emerged as an essential ecosystem residing within our body, which plays a significant role in development of immunity and homeostasis of the whole person. There are epidemiological studies suggesting decreased incidence of chronic lymphoid leukemia in children exposed to childhood infections, and increased incidence of Hodgkin’s lymphoma with high socioeconomic (higher cleanliness) status [75]. Furthermore, repeated exposure to antibiotic treatment has been associated with increased frequency of some cancers such as gastric, colorectal, lung, prostate and bladder cancers [76]. Germ-free conditions or treatment with broad-spectrum antibiotics in mice can result in slower progression of several tumor cell lines. In contrast, the same conditions can cause an accelerated growth of lung metastases when melanoma or non-small cell lung carcinoma cells are injected intravenously, suggesting that microbiota have both tumor-promoting and tumor-protective functions [77]. Using mice obtained from different sources, it has been clearly demonstrated that abundance or lack of a specific bacterial species in the microbiota significantly affects growth and progression of tumors [78]. Further, recent studies clearly suggest a prominent role played by microbiota in the failure or success of chemo- as well as immuno-therapeutics including those involving checkpoint inhibitors [78, 79]. Modulation of microbiota is emerging as a new approach to supplement chemo- and/or immunotherapy of various cancers (Fig. 4).

### Clinical application of immune and metabolic checkpoints for cancer therapy

**Fig. 5:**
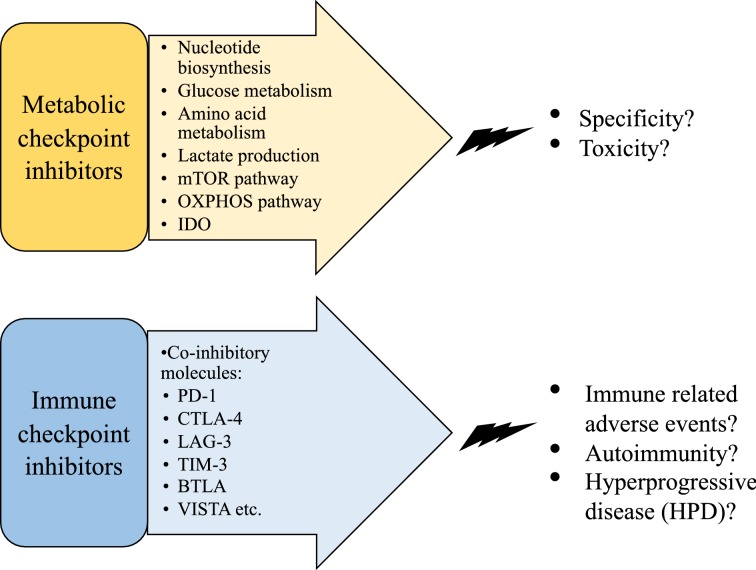
Clinical applications of immune and metabolic checkpoint inhibitors and their limitations. Various metabolic checkpoint inhibitors (top yellow panel) and immune checkpoint inhibitors (bottom blue panel) are being targeted for cancer therapy. However, while metabolic checkpoint inhibitors lack specificity against tumors resulting in toxicity to host cells, immune checkpoint inhibitors are associated with immune-related adverse events and exacerbation of cancer progression in some cases

Tumor-infiltrating lymphocytes (TILs) in the TME might be exploited clinically in eradicating the tumor from a host. However, it is evident that TIL function is adversely impacted or completely blunted by a variety of “metabolic and immune checkpoints”. Altered metabolic reprogramming of tumor cells has remained an attractive target for anticancer drug design and several chemotherapeutic drugs targeting specific metabolic pathway have emerged (Fig. 5). The metabolic pathway of nucleotide biosynthesis required for proliferation of tumor cells was the target for 5-fluorouracil, one of the most commonly used anticancer drugs [80]. Glucose metabolism of tumors has been targeted by 2-deoxyglucose (2-DG), an inhibitor of hexokinase that shuts down glycolysis, and by dichloroacetate (DCA), a metabolic checkpoint inhibitor inducing a shift from glycolysis to OXPHOS [81, 82]. Inhibition of tumor growth, and/or improving the efficacy of 5-fluorouracil can be accomplished by inhibiting lactate in the TME by inhibitors of lactate dehydrogenase (LDH) such as small molecule FX-11 or Galloflavin or by inhibiting the monocarboxylate transporter (MCT) with thalidomide, lenalidomide or pomalidomide [83]. Inhibition of the mTOR pathway by analogs of rapamycin has been approved for breast, renal and pancreatic cancers [84]. The diabetes drug metformin has shown some clinical efficacy as an anticancer drug by inhibiting the OXPHOS pathway [85]. It has been suggested that inhibition of IDO could be an important strategy to activate antitumor immunity by targeting tumor metabolism, however, clinical testing of experimental IDO inhibitors have shown disappointing results [86, 87]. The pathway for metabolism of glutamine, an amino acid required by fast dividing cancer cells, has been targeted by modified glutamine analogs as an anticancer strategy in preclinical studies [88]. However, non-specific toxicity presents a major hurdle of using these tumor checkpoint inhibitors for widespread clinical use, since all host cells use the same metabolic pathways for the survival. Further, activated T cells are especially vulnerable to metabolic checkpoint inhibitors since they have evolved strategies similar to tumor cells to satisfy their requirement of fast expansion upon activation.

This decade has seen tremendous growth in the development and clinical application of immune checkpoint inhibitor (ICI) therapy for various cancers (Fig. 5) and seven ICIs are currently approved by the FDA for treatment of various cancers [89]. The first clinically approved ICI was Ipilimumab, a monoclonal antibody (mAb) targeted against CTLA-4 for the treatment of advanced melanoma [90]. By blocking a prominent checkpoint molecule of T cells, this antibody blocks the T cell inhibition and promotes the activation of effector T cells that can then lead to killing of the tumor cells. PD-1 was the second checkpoint molecule targeted by ICIs, and the mAbs Pembrolizumab, Nivolumab and Cemiplimab are approved by the FDA for clinical use. The mAbs against PD-1 and its ligand PD-L1 (Atezolizumab, Avelumab and Durvalumab) are being used to treat a broad range of cancers, such as metastatic melanoma, renal-cell carcinoma (RCC), advanced non-small-cell lung cancer (NSCLC), Hodgkin’s lymphoma, bladder carcinoma, Merkel cell carcinoma, head and neck cancer, cutaneous squamous cell carcinoma (CSCC) and solid tumors [91, 92]. Furthermore, many clinical trials are on the way to expand the use of different mAbs against PD-1 and PD-L1 in a variety of cancers (https://clinicaltrials.gov). Significant clinical usage of the available ICIs has demonstrated that they are effective in only a fraction of treated patients (10–30%). Some patients develop resistance upon continuous use and more importantly there can be serious side effects to ICI use termed immune-related adverse events (irAEs). These can range in grades III-IV from severe to life-threatening. Since all of the checkpoint molecules are also part of homeostatic mechanisms of the immune system, irAEs are the result of disrupting this delicate homeostatic balance. Besides tumor-infiltrating or tumor antigen-specific T cells, T cells with various specificities and potentially autoimmune T cells can also be activated. The result can be inflammation and autoimmunity of various degrees [4].

Interestingly, treatment with ICIs not only inhibited the negative signaling in TILs, but also increased the glucose concentration within the TME, probably due to immune-mediated killing of tumor cells, and increased glycolytic activity and metabolic fitness of TILs, thereby also acting as metabolic checkpoint inhibitors [93]. Interestingly, some studies have also suggested that blocking PD-L1 has a direct effect on cancer cells themselves by reducing the glycolytic activity of tumor cells and increasing glucose levels available in the environment for the TILs [94]. These results suggest that there is potential in examining novel therapeutic approaches for cancer where inhibitors of immune and metabolic checkpoints are used strategically to enhance overall anti-tumor immune responses. Although this idea is theoretically attractive, a recent clinical trial testing the combination of anti-PD-1 mAb and an experimental IDO inhibitor resulted in disappointing clinical responses that terminated the trial [87]. It is not clear whether this failure was due to insufficient efficacy of the tested IDO inhibitor, or the approach overall.

The availability of ICIs has also rejuvenated interest in therapeutic vaccines for cancers. The idea, as well as the candidate vaccines, were shelved due to insufficient efficacy in the 1990s and 2000s. Now it seems plausible that tumor-specific T cells can be primed and stimulated against tumor antigens, provided that the inhibitory brakes on them have been relieved by the use of ICIs [95].

Besides CTLA-4 and PD-1/PD-L1 targeted mAbs, development of mAbs against other checkpoint molecules of T cells, e.g., TIM-3, LAG-3 and TIGIT, and various combinations of ICI mAbs are also underway for the treatment of various cancers [96]. Time will tell whether combining various ICIs for cancer treatment will provide enhanced efficacy and minimization of adverse events.

In this regard, a multi-functional pan-adenocarcinoma associated molecule MUC1 appears to be an excellent target for inhibition by mAbs, which could potentially act as inhibitor of metabolic checkpoint in tumor cells and immune checkpoint on T cells, while additionally blocking invasiveness and metastasis of tumor cells bearing MUC1 [69, 72, 73, 97]. Along with these plausible mechanisms, mAb binding to tumor-expressed MUC1 could lead to their enhanced lysis through ADCC (antibody dependent cellular cytotoxicity) and enhanced presentation by APCs, resulting in efficient stimulation of tumor-specific T cells. Currently, clinical trials of anti-MUC1 mAbs are underway for cancer treatment (https://clinicaltrials.gov). However, their efficacy and effector mechanisms remain to be examined.

Detailed investigations of low response rate and serious irAEs related to anti-PD-1 mAb treatment in patients also demonstrated a unique link between the gut microbiota and ICI treatment [98]. Using germ-free mice transplanted with fecal microbiota from anti-PD-1 responder or non-responder patients, it has been clearly demonstrated that addition of certain bacterial strains in the gut of mice leads to better anti-tumor response rates of anti-PD-1/PD-L1 therapy [99]. Therefore, a short-term future trajectory of cancer therapy would favor the combining of ICIs with microbiome-modulating regimens. To this end, several clinical trials are underway to test whether treatment with ICIs along with probiotics, fecal microbiota transfer (FMT, either as a whole or as a selected bacterial mix), and/or diet-modification would result in enhanced response rates and reduced adverse events (https://clinicaltrials.gov).

In addition to serious irAEs reported with ICI, an emerging but serious clinical observation has been defined as hyperprogressive disease (HPD). This is a more aggressive cancer progression following ICI treatment in a significant proportion of patients. HPD has been observed in patients with melanoma, colorectal cancer, non-small cell lung carcinoma, head and neck cancer, ovarian cancer and lymphoma upon treatment with anti-PD-1/PD-L1 mAb therapy [100]. These observations acknowledge serious clinical manifestations in multiple cancers, whereby treatment with ICIs is associated with worsening of cancer disease. The exact mechanism of HPD upon ICI treatment is not clear yet but could be paradoxically attributed to increased inflammation or increased immune suppression (due to mAb acting as agonists of checkpoints). It is possible that microbiota-based homeostatic control of aberrant immune responses may provide a tangible solution to improve the clinical response and management of adverse events with ICI therapy.

## Conclusions

The idea of killing and eliminating tumors by mobilizing the host’s own immune system has been around for more than half a century, initially brought forward as active specific immunotherapy (ASI) for cancers, which resulted in clinical testing of numerous therapeutic cancer vaccines. However, their clinical failure gave rise to our current understanding of the metabolic- and immune-related checkpoints (brakes) or inhibitory molecules, the specific micro-environment present in the tumors, and ways to release those brakes with ICIs. ICIs, although considered as breakthrough cancer therapy of the current decade, still suffer from limited response rates, development of resistance, serious irAEs, and most seriously hyperprogressive disease. The future course of cancer therapy research and therapeutic regimens will depend on our expanding knowledge of various immune and metabolic checkpoints, and our increasing understanding of the microbiota and their impact on immunotherapy.

## Data Availability

Not applicable.

## Abbreviations

2-DG: 2-deoxyglucose
A2A: adenosine receptor
ADCC: antibody dependent cellular cytotoxicity
APCs: antigen presenting cells
ASI: active specific immunotherapy
BTLA: B and T lymphocyte attenuator
CAF: cancer associated fibroblasts
CEA: carcinoembryonic antigen
CSC: cancer stem cell
CSCC: cutaneous squamous cell carcinoma
CT: cytoplasmic tail
CTLA-4: cytotoxic T lymphocyte associated antigen-4
DCA: dichloroacetate
ECM: extracellular matrix
EGFR: epithelial growth factor receptor
EMT: epithelial to mesenchymal transition
FAO: fatty acid oxidation
FMT: fecal microbiota transfer
HBV: hepatitis B virus
HCV: hepatitis C virus
HGF: hepatocyte growth factor
HIF-1α: hypoxia induced factor-1α
HPD: hyper progressive disorder
Hsp: heat shock protein
ICI: immune checkpoint inhibitor
IDO: indolamine 2,3-dioxygenase
IFN: interferon
IGF-1: insulin like growth factor-1
IL-1: interleukin-1
irAEs: immune-related adverse events
ITAM: immunotyrosine-based activation motif
ITIM: immunotyrosine-based inhibitory motif
LAG-3: lymphocyte activation gene-3
LDH: lactate dehydrogenase
MCP-1: monocyte chemotactic protein-1
MCT: monocarboxylate transporter
MDSC: myeloid derived suppressor cells
MMP: matrix metalloprotein
mTOR: mammalian target of rapamycin
MUC1: MUC1 mucin
NK: natural killer
NSCLC: non-small cell lung cancer
OXPHOS: oxidative phosphorylation
PD-1: programmed cell death-1
PD-L1: programmed cell death ligand-1
PEM: polymorphic epithelial mucin
PI3 K-1: phosphoinositide-dependent protein kinase-1
PSA: prostate specific antigen
RCC: renal cell carcinoma
SDF1: stromal cell derived factor 1
SIGLEC: sialic acid binding immunoglobulin type lectin
TAAs: tumor associated antigens
TGF-β: tumor growth factor-beta
TILs: tumor infiltrating lymphocytes
TIM-3: T cell immunoglobulin and mucin domain-1
TLR: toll like receptor
TME: tumor microenvironment
TNF: tumor necrosis factor
T_reg_: regulatory T cells
VEGF: vascular endothelial growth factor
VISTA: V-domain Ig suppressor of T cell activation
VNTR: variable number of tandem repeat
WAT: white adipose tissue

## References

[1] International Agency for Research on Cancer (2018) Latest global cancer data: cancer burden rises to 18.1 million new cases and 9.6 million cancer deaths in 2018. Press Release, pp 1–3. http://gco.iarc.fr/. Accessed 12 Sept 2018

[2] Society AC (2018) Cancer facts and figures 2018. American Cancer Society

[3] Siegel RL, Miller KD, Jemal A (2019). Cancer statistics, 2019. CA Cancer J Clin..

[4] La-Beck NM, Jean GW, Huynh C, Alzghari SK, Lowe DB (2015). Immune checkpoint inhibitors: new insights and current place in cancer therapy. Pharmacotherapy.

[5] Bodey B, Bodey B, Siegel SE, Kaiser HE (2000). Failure of cancer vaccines: the significant limitations of this approach to immunotherapy. Anticancer Res.

[6] Buonaguro L, Petrizzo A, Tornesello ML, Buonaguro FM (2011). Translating tumor antigens into cancer vaccines. Clin Vaccine Immunol.

[7] Wang M, Zhao J, Zhang L, Wei F, Lian Y, Wu Y (2017). Role of tumor microenvironment in tumorigenesis. J Cancer.

[8] Lazebnik Y (2010). What are the hallmarks of cancer?. Nat Rev Cancer.

[9] Baum J, Duffy HS (2011). Fibroblasts and myofibroblasts: what are we talking about?. J Cardiovasc Pharmacol.

[10] Yu Y, Xiao C-H, Tan L-D, Wang Q-S, Li X-Q, Feng Y-M (2014). Cancer-associated fibroblasts induce epithelial–mesenchymal transition of breast cancer cells through paracrine TGF-β signalling. Br J Cancer.

[11] Franco OE, Shaw AK, Strand DW, Hayward SW (2010). Cancer associated fibroblasts in cancer pathogenesis. Semin Cell Dev Biol.

[12] Calon A, Tauriello DVF, Batlle E (2014). TGF-beta in CAF-mediated tumor growth and metastasis. Semin Cancer Biol.

[13] Olumi AF, Grossfeld GD, Hayward SW, Carroll PR, Tlsty TD, Cunha GR (1999). Carcinoma-associated fibroblasts direct tumor progression of initiated human prostatic epithelium. Cancer Res.

[14] Kalluri R (2016). The biology and function of fibroblasts in cancer. Nat Rev Cancer.

[15] Frantz C, Stewart KM, Weaver VM (2010). The extracellular matrix at a glance. J Cell Sci.

[16] Lu P, Weaver VM, Werb Z (2012). The extracellular matrix: a dynamic niche in cancer progression. J Cell Biol.

[17] Hida K, Ohga N, Kurosu T, Totsuka Y, Shindoh M (2010). Crosstalk between blood vessels and tumor microenvironment. Oral Sci Int.

[18] Distler JHW, Hirth A, Kurowska-Stolarska M, Gay RE, Gay S, Distler O (2003). Angiogenic and angiostatic factors in the molecular control of angiogenesis. Q J Nucl Med.

[19] Petrova V, Annicchiarico-Petruzzelli M, Melino G, Amelio I (2018). The hypoxic tumour microenvironment. Oncogenesis.

[20] Li Z, Rich JN (2010). Hypoxia and hypoxia inducible factors in cancer stem cell maintenance. Curr Top Microbiol Immunol.

[21] Gajewski TF, Schreiber H, Fu Y-X (2013). Innate and adaptive immune cells in the tumor microenvironment. Nat Immunol.

[22] Kusmartsev S, Gabrilovich DI (2006). Effect of tumor-derived cytokines and growth factors on differentiation and immune suppressive features of myeloid cells in cancer. Cancer Metastasis Rev.

[23] Grivennikov SI, Greten FR, Karin M (2010). Immunity, inflammation, and cancer. Cell.

[24] Martínez A, Urios A, Felipo V, Blanco M (2001). Mutagenicity of nitric oxide-releasing compounds in *Escherichia coli*: effect of superoxide generation and evidence for two mutagenic mechanisms. Mutat Res Toxicol Environ Mutagen.

[25] Elinav E, Nowarski R, Thaiss CA, Hu B, Jin C, Flavell RA (2013). Inflammation-induced cancer: crosstalk between tumours, immune cells and microorganisms. Nat Rev Cancer.

[26] Brayman M, Thathiah A, Carson DD (2004). MUC1: a multifunctional cell surface component of reproductive tissue epithelia. Reprod Biol Endocrinol.

[27] Duong MN, Geneste A, Fallone F, Li X, Dumontet C, Muller C (2017). The fat and the bad: mature adipocytes, key actors in tumor progression and resistance. Oncotarget..

[28] Greenberg AS, Obin MS (2006). Obesity and the role of adipose tissue in inflammation and metabolism. Am J Clin Nutr.

[29] Amemori S, Ootani A, Aoki S, Fujise T, Shimoda R, Kakimoto T (2007). Adipocytes and preadipocytes promote the proliferation of colon cancer cells in vitro. Am J Physiol Gastrointest Liver Physiol.

[30] Vazquez Rodriguez G, Abrahamsson A, Jensen LDE, Dabrosin C (2018). Adipocytes promote early steps of breast cancer cell dissemination via interleukin-8. Front Immunol..

[31] Takahara K, Ii M, Inamoto T, Komura K, Ibuki N, Minami K (2014). Adipose-derived stromal cells inhibit prostate cancer cell proliferation inducing apoptosis. Biochem Biophys Res Commun.

[32] Ruf M, Moch H, Schraml P (2016). PD-L1 expression is regulated by hypoxia inducible factor in clear cell renal cell carcinoma. Int J Cancer.

[33] Vaupel Peter, Mayer Arnulf (2016). Hypoxia-Driven Adenosine Accumulation: A Crucial Microenvironmental Factor Promoting Tumor Progression. Advances in Experimental Medicine and Biology.

[34] Ohta A (2016). A metabolic immune checkpoint: adenosine in tumor microenvironment. Front Immunol..

[35] Sugiura A, Rathmell JC (2018). Metabolic barriers to T cell function in tumors. J Immunol..

[36] Colegio OR, Chu N-Q, Szabo AL, Chu T, Rhebergen AM, Jairam V (2014). Functional polarization of tumour-associated macrophages by tumour-derived lactic acid. Nature..

[37] Brand A, Singer K, Koehl GE, Kolitzus M, Schoenhammer G, Thiel A (2016). LDHA-associated lactic acid production blunts tumor immunosurveillance by T and NK cells. Cell Metab.

[38] Renner K, Singer K, Koehl GE, Geissler EK, Peter K, Siska PJ (2017). Metabolic hallmarks of tumor and immune cells in the tumor microenvironment. Front Immunol.

[39] Uyttenhove C, Pilotte L, Théate I, Stroobant V, Colau D, Parmentier N (2003). Evidence for a tumoral immune resistance mechanism based on tryptophan degradation by indoleamine 2,3-dioxygenase. Nat Med.

[40] Delgoffe GM, Kole TP, Zheng Y, Zarek PE, Matthews KL, Xiao B (2009). The mTOR kinase differentially regulates effector and regulatory T cell lineage commitment. Immunity.

[41] Gerriets VA, Kishton RJ, Johnson MO, Cohen S, Siska PJ, Nichols AG (2016). Foxp3 and toll-like receptor signaling balance Treg cell anabolic metabolism for suppression. Nat Immunol.

[42] Sun I-H, Oh M-H, Zhao L, Patel CH, Arwood ML, Xu W (2018). mTOR complex 1 signaling regulates the generation and function of central and effector Foxp3 + regulatory T cells. J Immunol..

[43] Phan AT, Goldrath AW (2015). Hypoxia-inducible factors regulate T cell metabolism and function. Mol Immunol..

[44] Lim S, Liu H, da Silva LM, Arora R, Liu Z, Phillips JB (2016). Immunoregulatory protein B7-H3 reprograms glucose metabolism in cancer cells by ROS-mediated stabilization of HIF1α. Cancer Res.

[45] Sun C, Mezzadra R, Schumacher TN (2018). Regulation and function of the PD-L1 checkpoint. Immunity.

[46] Varn FS, Wang Y, Mullins DW, Fiering S, Cheng C (2017). Systematic pan-cancer analysis reveals immune cell interactions in the tumor microenvironment. Cancer Res.

[47] Smith-Garvin JE, Koretzky GA, Jordan MS (2009). T cell activation. Annu Rev Immunol..

[48] Wingren AG, Parra E, Varga M, Kalland T, Sjogren HO, Hedlund G (1995). T cell activation pathways: B7, LFA-3, and ICAM-1 shape unique T cell profiles. Crit Rev Immunol.

[49] Watanabe N, Nakajima H (2012). Coinhibitory molecules in autoimmune diseases. Clin Dev Immunol.

[50] Chen L, Flies DB (2013). Molecular mechanisms of T cell co-stimulation and co-inhibition. Nat Rev Immunol.

[51] Stanford SM, Rapini N, Bottini N (2012). Regulation of TCR signalling by tyrosine phosphatases: from immune homeostasis to autoimmunity. Immunology.

[52] Buchbinder EI, Desai A (2016). CTLA-4 and PD-1 pathways similarities, differences, and implications of their inhibition. Am J Clin Oncol Cancer Clin Trials.

[53] Tivol EA, Borriello F, Schweitzer AN, Lynch WP, Bluestone JA, Sharpe AH (1995). Loss of CTLA-4 leads to massive lymphoproliferation and fatal multiorgan tissue destruction, revealing a critical negative regulatory role of CTLA-4. Immunity.

[54] Nishimura H, Honjo T (2001). PD-1: an inhibitory immunoreceptor involved in peripheral tolerance. Trends Immunol.

[55] Anderson AC, Joller N, Kuchroo VK (2016). Lag-3, Tim-3, and TIGIT: co-inhibitory receptors with specialized functions in immune regulation. Immunity.

[56] Howie D, Simarro M, Sayos J, Guirado M, Sancho J, Terhorst C (2002). Molecular dissection of the signaling and co-stimulatory functions of CD150 (SLAM): CD150/SAP binding and CD150-mediated costimulation. Blood.

[57] Legat A, Speiser DE, Pircher H, Zehn D, Fuertes Marraco SA (2013). Inhibitory receptor expression depends more dominantly on differentiation and activation than “exhaustion” of human CD8 T cells. Front Immunol.

[58] Wherry EJ, Kurachi M (2015). Molecular and cellular insights into T cell exhaustion. Nat Rev Immunol.

[59] Wherry EJ (2011). T cell exhaustion. Nat Immunol.

[60] Schietinger A, Greenberg PD (2014). Tolerance and exhaustion: defining mechanisms of T cell dysfunction. Trends Immunol..

[61] Chang C-H, Qiu J, O’Sullivan D, Buck MD, Noguchi T, Curtis JD (2015). Metabolic competition in the tumor microenvironment is a driver of cancer progression. Cell.

[62] Chaudhary B, Elkord E (2016). Regulatory T cells in the tumor microenvironment and cancer progression: role and therapeutic targeting. Vaccines.

[63] Hattrup CL, Gendler SJ (2008). Structure and function of the cell surface (tethered) mucins. Annu Rev Physiol.

[64] Treon SP, Maimonis P, Bua D, Young G, Raje N, Mollick J (2000). Elevated soluble MUC1 levels and decreased anti-MUC1 antibody levels in patients with multiple myeloma. Blood.

[65] Nath S, Mukherjee P (2014). MUC1: a multifaceted oncoprotein with a key role in cancer progression. Trends Mol Med.

[66] Apostolopoulos V, Stojanovska L, Gargosky SE (2015). MUC1 (CD227): a multi-tasked molecule. Cell Mol Life Sci.

[67] Poh TW, Bradley JM, Mukherjee P, Gendler SJ (2009). Lack of Muc1-regulated beta-catenin stability results in aberrant expansion of CD11b+ Gr1+ myeloid-derived suppressor cells from the bone marrow. Cancer Res.

[68] Jonckheere N, Van Seuningen I (2010). The membrane-bound mucins: from cell signalling to transcriptional regulation and expression in epithelial cancers. Biochimie..

[69] Mehla K, Singh PK (2014). MUC1: a novel metabolic master regulator. Biochim Biophys Acta Rev Cancer..

[70] Agrawal B, Krantz MJ, Parker J, Longenecker BM (1998). Expression of MUC1 mucin on activated human T cells: implications for a role of MUC1 in normal immune regulation. Cancer Res.

[71] Agrawal B, Krantz MJ, Reddish MA, Longenecker BM (1998). Cancer-associated MUC1 mucin inhibits human T-cell proliferation, which is reversible by IL-2. Nat Med.

[72] Konowalchuk JD, Agrawal B (2012). MUC1 mucin is expressed on human T-regulatory cells: function in both co-stimulation and co-inhibition. Cell Immunol..

[73] Konowalchuk JD, Agrawal B (2012). MUC1 is a novel costimulatory molecule of human T cells and functions in an AP-1-dependent manner. Hum Immunol.

[74] Wykes M, MacDonald KPA, Tran M, Quin RJ, Xing PX, Gendler SJ (2002). MUC1 epithelial mucin (CD227) is expressed by activated dendritic cells. J Leukoc Biol..

[75] Oikonomopoulou K, Brinc D, Kyriacou K, Diamandis EP (2013). Infection and cancer: revaluation of the hygiene hypothesis. Clin Cancer Res.

[76] Boursi B, Mamtani R, Haynes K, Yang Y-X (2015). Recurrent antibiotic exposure may promote cancer formation—another step in understanding the role of the human microbiota?. Eur J Cancer..

[77] Goodman B, Gardner H (2018). The microbiome and cancer. J Pathol.

[78] American Association for Cancer Research (2016). The efficacy of cancer immunotherapy relies on gut microbiota. Cancer Discov.

[79] Gopalakrishnan V, Helmink BA, Spencer CN, Reuben A, Wargo JA (2018). The influence of the gut microbiome on cancer, immunity, and cancer immunotherapy. Cancer Cell.

[80] Longley DB, Harkin DP, Johnston PG (2003). 5-Fluorouracil: mechanisms of action and clinical strategies. Nat Rev Cancer.

[81] Pelicano H, Martin DS, Xu RH, Huang P (2006). Glycolysis inhibition for anticancer treatment. Oncogene.

[82] Granchi C, Minutolo F (2012). Anticancer agents that counteract tumor glycolysis. ChemMedChem.

[83] Doherty JR, Cleveland JL (2013). Targeting lactate metabolism for cancer therapeutics. J Clin Investig.

[84] Meric-Bernstam F, Gonzalez-Angulo AM (2009). Targeting the mTOR signaling network for cancer therapy. J Clin Oncol.

[85] Daugan M, Dufaÿ Wojcicki A, D’Hayer B, Boudy V (2016). Metformin: an anti-diabetic drug to fight cancer. Pharmacol Res.

[86] Prendergast GC, Malachowski WP, DuHadaway JB, Muller AJ (2017). Discovery of IDO1 inhibitors: from bench to bedside. Can Res.

[87] Komiya T, Huang CH (2018). Updates in the clinical development of epacadostat and other indoleamine 2,3-dioxygenase 1 inhibitors (IDO1) for human cancers. Front Oncol.

[88] Lukey MJ, Wilson KF, Cerione RA (2013). Therapeutic strategies impacting cancer cell glutamine metabolism. Fut Med Chem.

[89] Abril-Rodriguez G, Ribas A (2017). SnapShot: immune checkpoint inhibitors. Cancer Cell.

[90] Wolchok JD, Hodi FS, Weber JS, Allison JP, Urba WJ, Robert C (2013). Development of ipilimumab: a novel immunotherapeutic approach for the treatment of advanced melanoma. Ann N Y Acad Sci.

[91] Fuertes Marraco SA, Neubert NJ, Verdeil G, Speiser DE (2015). Inhibitory receptors beyond T cell exhaustion. Front Immunol.

[92] Pardoll DM (2012). The blockade of immune checkpoints in cancer immunotherapy. Nat Rev Cancer.

[93] Lim S, Phillips JB, Madeira da Silva L, Zhou M, Fodstad O, Owen LB (2017). Interplay between immune checkpoint proteins and cellular metabolism. Cancer Res.

[94] Qorraj M, Böttcher M, Mougiakakos D (2017). PD-L1/PD-1: new kid on the “immune metabolic” block. Oncotarget.

[95] Collins JM, Redman JM, Gulley JL (2018). Combining vaccines and immune checkpoint inhibitors to prime, expand, and facilitate effective tumor immunotherapy. Expert Rev Vaccines..

[96] Zahavi D, Weiner L (2019). Targeting multiple receptors to increase checkpoint blockade efficacy. Int J Mol Sci.

[97] McDermott KM, Crocker PR, Harris A, Burdick MD, Hinoda Y, Hayashi T (2001). Overexpression of MUC1 reconfigures the binding properties of tumor cells. Int J Cancer.

[98] Sivan A, Corrales L, Hubert N, Williams JB, Aquino-Michaels K, Earley ZM (2015). Commensal *Bifidobacterium* promotes antitumor immunity and facilitates anti-PD-L1 efficacy. Science..

[99] Gopalakrishnan V, Spencer CN, Nezi L, Reuben A, Andrews MC, Karpinets TV (2018). Gut microbiome modulates response to anti-PD-1 immunotherapy in melanoma patients. Science..

[100] Champiat S, Ferrara R, Massard C, Besse B, Marabelle A, Soria J-C (2018). Hyperprogressive disease: recognizing a novel pattern to improve patient management. Nat Rev Clin Oncol..

